# Development of a Preclinical Therapeutic Model of Human Brain Metastasis with Chemoradiotherapy

**DOI:** 10.3390/ijms14048306

**Published:** 2013-04-16

**Authors:** Antonio Martínez-Aranda, Vanessa Hernández, Cristina Picón, Ignasi Modolell, Angels Sierra

**Affiliations:** 1 Biological Clues of the Invasive and Metastatic Phenotype Group, Bellvitge Biomedical Research Institute (IDIBELL), L’ Hospitalet de Llobregat, Barcelona 08907, Spain; E-Mails: amartinez@idibell.cat (A.M.-A.); vhernanadez@idibell.cat (V.H.); 2 Autonoma University of Barcelona (UAB), Faculty of Biosciences, Campus Bellaterra, Building C, Cerdanyola del Vallés, Barcelona 08193, Spain; 3 Medical Physics Service, Oncology Catalan Institut, Duran I Reynals Hospital, L’Hospitalet de Llobregat, Barcelona 08907, Spain; E-Mails: cpicon@iconcologia.net (C.P.); imodolell@iconcologia.net (I.M.)

**Keywords:** brain metastasis, breast cancer, experimental models, radiation, temozolomide, therapy

## Abstract

Currently, survival of breast cancer patients with brain metastasis ranges from 2 to 16 months. In experimental brain metastasis studies, only 10% of lesions with the highest permeability exhibited cytotoxic responses to paclitaxel or doxorubicin. Therefore, radiation is the most frequently used treatment, and sensitizing agents, which synergize with radiation, can improve the efficacy of the therapy. In this study we used 435-Br1 cells containing the fluorescent protein (*eGFP*) gene and the photinus luciferase (*PLuc*) gene to develop a new brain metastatic cell model in mice through five *in vivo*/*in vitro* rounds. BR-eGFP-CMV/Luc-V5 brain metastatic cells induce parenchymal brain metastasis within 60.8 ± 13.8 days of intracarotid injection in all mice. We used this model to standardize a preclinical chemoradiotherapy protocol comprising three 5.5 Gy fractions delivered on consecutive days (overall dose of 16.5 Gy) which improved survival with regard to controls (60.29 ± 8.65 *vs.* 47.20 ± 11.14). Moreover, the combination of radiotherapy with temozolomide, 60 mg/Kg/day orally for five consecutive days doubled survival time of the mice 121.56 ± 52.53 days (Kaplan-Meier Curve, *p* < 0.001). This new preclinical chemoradiotherapy protocol proved useful for the study of radiation response/resistance in brain metastasis, either alone or in combination with new sensitizing agents.

## 1. Introduction

Although breast cancer metastasis to either the brain parenchyma or the leptomeninges is generally a late feature of the disease, metastasis to the central nervous system (CNS) is associated with a dismal prognosis [1] and causes significant morbidity and mortality in breast cancer patients [2]. Indeed, 30%–40% of patients with disseminated breast carcinoma develop metastasis in the CNS, with survival ranging from 2 to 16 months [3].

The high rate of CNS metastasis may be related to the greater survival in patients receiving chemotherapy and to the difficulty that current systemic treatments have in overcoming the blood brain barrier (BBB) [3–5]. The amplification of the ErbB2 receptor tyrosine kinase or triple negative tumors (negative estrogen and progesterone receptors and normal ErbB2 expression) increases the incidence of brain metastases, which may exceed 30% of patients [6,7].

The mechanistic process in the pathogenesis of brain metastasis is complex because cancer cells have to cross the blood brain barrier (BBB) located in the brain vascular endothelium [8]. The brain places different demands on the invading tumor cells, which are obliged to establish glial interactions in order to colonize it [9]. Many issues such as the mechanism of arrest and the role of cell extravasation, angiogenesis, and dormancy remain controversial [10]. The essential step takes place at the vascular branch point, where the persistent close contact between metastatic cells and microvessels induces perivascular growth via vessel cooption [11]. The extracellular matrix, pericytes and astrocyte foot processes mediate the impermeability of the BBB, which is increased by the high electrical resistance in brain capillaries hindering the entrance of polar and ionic substrates [12].

The BBB remains a significant impediment to the delivery and efficacy of standard chemotherapy for brain metastasis of breast cancer [13]. Experimental brain metastasis studies showed that most metastases exhibit increased BBB permeability, which is poorly correlated with lesion size, and that only 10% of lesions with the highest permeability exhibited cytotoxic responses to paclitaxel or doxorubicin [14]. This evidence reinforces the need for brain-permeable molecular therapeutics and radiation-sensitizing agents able to synergize with radiation therapy, thus improving the treatment of established brain metastases and minimizing the cognitive losses suffered by a proportion of patients after radiotherapy [15,16].

In this scenario, models mimicking brain metastasis are needed to examine the pathogenesis in more depth and to develop experimental therapeutic approaches able to improve our understanding of antimetastatic activity and toxicity of numerous experimental agents [17,18]. The major problem is that tumor foci occasionally grow in the brain and successive rounds of systemic injections are needed to increase the propensity of breast cancer cells to metastasize in brain [19]. Schackert and Fidler (1988) described an animal model of brain metastasis, based on intracarotid (*CA*) injection of human cell lines in *nude mice*[20], which they used to characterize melanoma brain metastasis and various types of carcinoma [13]. In earlier work we modified this method, which progressed within a 20 to 62-day time window post–injection in 69% of cases, as detected by *in vivo* MR and further confirmed by the histological analysis of samples [21].

Mouse models of breast cancer and advanced metastatic disease are needed in order to be able to carry out preclinical therapeutic studies. Since radiation therapy is the most commonly used procedure for the treatment of brain metastasis, we used triple negative 435-Br1 cells containing the fluorescent protein (*eGFP*) gene and the photinus luciferase (*PLuc*) gene, in order to develop a new brain metastatic cell variant which induced parenchymal brain metastasis within a 60-day time window post-injection in all cases. BR-eGFP-CMV/Luc cells were injected in the left ventricle and their further isolation from mouse brain was repeated five times, obtaining BR-eGFP-CMV/LucV5 cells through these cycles (BRV5).

Temozolomide (TMZ) has been broadly used in glioblastoma experimental therapeutic models and in clinical settings, objectivizing regression or delay of tumor progression [22–25]. Studies in patients show that association of whole brain radiation plus TMZ for brain metastasis treatment is well tolerated [26]. As an oral anticancer agent, has a favorable toxicity and pharmacokinetic profile, allowing its clinical investigation for brain metastasis from solid tumors in combination with other treatments, such as radiotherapy [27]. Using BRV5 cells and BRV5CA1 cells (obtained from brain metastasis after intracarotid, *IC*, injection of BRV5 cells), we standardized a preclinical protocol combining radiotherapy and TMZ, as radiosensitizer, that can be used to assess new drugs and/or new radiation protocols to combat breast cancer brain metastasis. This versatile model mimics triple negative breast cancer clinical brain metastasis growth, which can be analyzed *in vivo* when mice are submitted to different therapeutic protocols.

## 2. Results

### 2.1. BR-eGFP-CMV/Luc-V5 Brain Metastatic Cells

**Figure 1:**
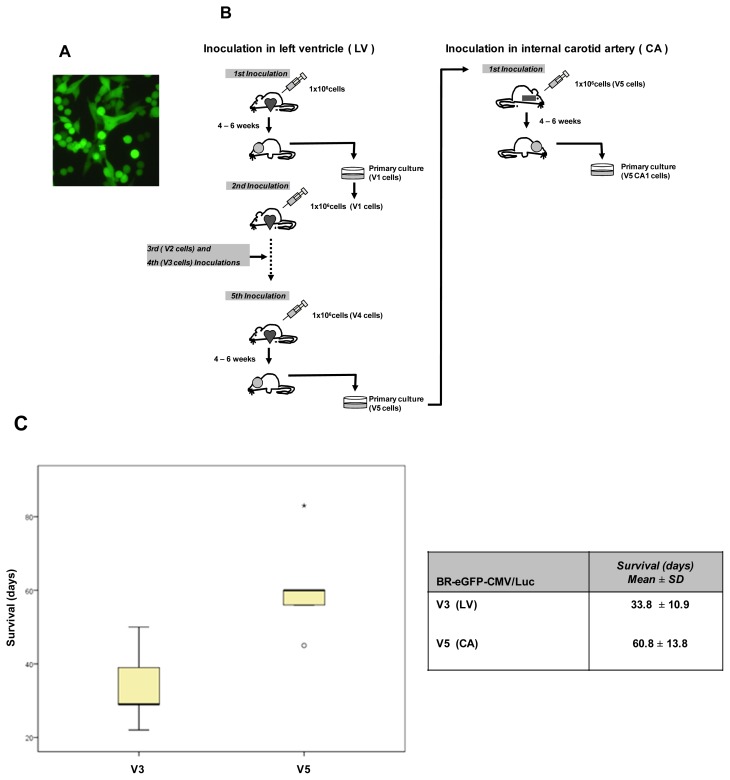
BR-eGFP-CMV/Luc brain metastatic cells. (**A**) Cells viewed under fluorescence microscopy in culture (20×); (**B**) Flow work chart of Inoculation of BR-eGFP-CMV/Luc cells in left ventricle. The diagram shows the steps followed to obtain cells that have been inoculated five times in the left ventricle of female athymic mice. The cells obtained from the brain when mice were injected for the first time in the left ventricle are named V1 cells, and those obtained after the fifth injection are named V5 cells (BRV5 cells). Further intracarotid injection experiments were performed using these V5 cells and then isolated from brain tissue (BRV5CA1 cells); (**C**) Statistical analysis comparing survival of mice injected with BRV3 and BRV5 cells (Mann-Whitney Test, 2-tailed, *p* = 0.016).

A cell population that uniformly expressed the highest levels of eGFP (BR-eGFP-CMV/Luc) was selected by FACS and was used as a starting point for the selection of more specific brain metastatic cells (Figure 1A). We started by injecting BR-eGFP-CMV/Luc cells into the left ventricle and continued until five *in vivo*/*in vitro* rounds, before intracarotid injection (Figure 1B).

**Figure 2:**
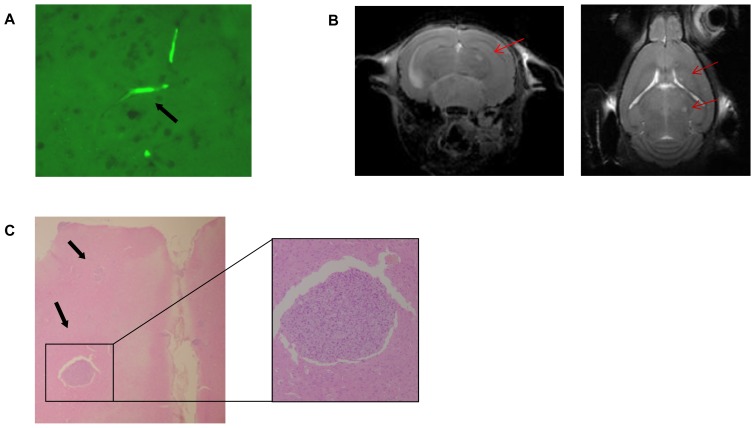
Histological view of cells inoculated in mice by intracarotid injection. (**A**) 20 μm slice from CD1 female mouse brain 5 min after intracarotid injection of cells seen under fluorescence microscope (40×). Note the fluorescence from the inoculated cells in the lumen of the brain vessels (see black arrow); (**B**) Magnetic resonance imaging scans (T2w and high resolution sequences) on coronal (**left**) and axial (**right**) planes respectively from a mouse skull 29 days post-injection of BR-eGFP-CMV/Luc (V5CA1) cells. In both scans several small lesions can be seen in the right hemisphere of the brain (see red arrows); (**C**) H&E staining of a 5 μm histological slice (4×) from right brain hemisphere 30 days post-injection in the right carotid artery of BR-eGFP-CMV/Luc (V5CA1) cells. Note the two metastases of different sizes (see black arrows). On the right is the largest metastasis at (20×).

435-Br1 cells have been the mainstay of experimental brain metastasis via infusion into either the carotid artery or the left cardiac ventricle [20,28]. In previous work we reported that 435Br1 metastatic cells have a heterogeneous distribution, secondary to the entry points of the cells in the brain parenchyma, which was confirmed by the histological analysis of *ex vivo* samples [21]. Five minutes after *CA* injection of BRV5 cells their presence in vessels was confirmed by fluorescence microscopy, which identified them in the lumen of the intraparenchymal brain arteries (see Figure 2A). These results are in agreement with reports of a strong bioluminescence signal immediately after carotid injection of MDA-BM-435 cells, which was detected in the hemisphere of the brain and persisted at the same level of intensity through days 5 to 7 before the exponential increase [29].

The fourth round we injected BR-eGFP-CMV/Luc-V3 (BRV3) cells into the left ventricle inducing mainly mediastinal lymph node metastases that killed five out of five mice 33.8 ± 10.9 days after cells inoculation. Fluorescent cells were found in the brain of two of five mice, in the lungs in five, in the liver in four, in the ovaries in two, in suprarenal glands in one and in mediastinal lymph nodes in five (Table 1). BR-eGFP-CMV/Luc-V5 (BRV5) cells that were injected intracarotid induced symptoms of brain disabilities in five mice, consisting in lateralization of the movements and asymmetry in addition to loss of weight. We found fluorescent cells in the brain of all five mice studied, in the lungs in five, in the liver in one, in the ovaries in two, in suprarenal glands in three and in mediastinal lymph nodes in three (Table 1). All mice showed symptoms of brain disabilities with a median of survival of 60.8 ± 13.8 days after cells inoculation (Figure 1C). Since the mice died without symptoms of breathing difficulties and without macroscopic metastasis in lungs or lymph nodes, the brain metastasis observed in histological slides indicated that brain metastasis progression was the cause of death. Differences in the survival evolution of mice inoculated with BRV3 cells with regard to those inoculated with BRV5 cells were statistically significant (Mann-Whitney Test, 2-tailed *p* = 0.016).

The perioperative mortality following *LV* injection was 11% *vs.* 8% in mice in which brain metastases were induced via *IC.*

We used MRI for *in vivo* visualization of the parenchymal location of brain metastases (Figure 2B). As expected, the longitudinal studies of metastatic progression in coronal (Figure 2B left) and axial sections (Figure 2B right) identified metastasis mainly in the brain parenchyma. After inoculation in the right carotid artery, MRI scans (T2w and high resolution sequences) on coronal and axial planes were acquired weekly. Images obtained at day 7, 14 and 21 post-inoculation showed no abnormal signal in the brain, and no clinical symptoms were observed. At day 29 when the animals showed some external signs of illness, such as a slight increase in the size of the right eye and hypoactivity, MRI showed small lesions in the right hemisphere of the brain. Post-mortem histological analyses in transversal and coronal slices were carried out in most cases to confirm the extension of the disease, staining the slides with hematoxylin and eosin (Figure 2C).

### 2.2. The Therapeutic Irradiation Model

**Figure 3:**
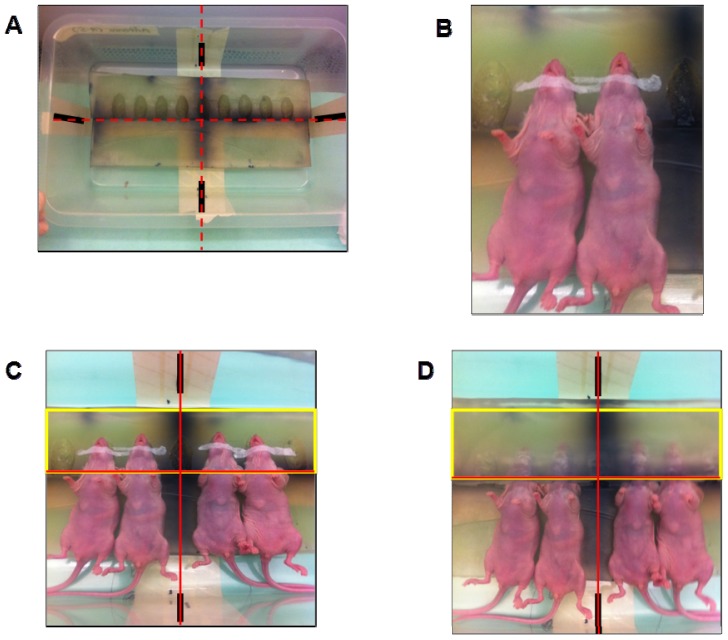

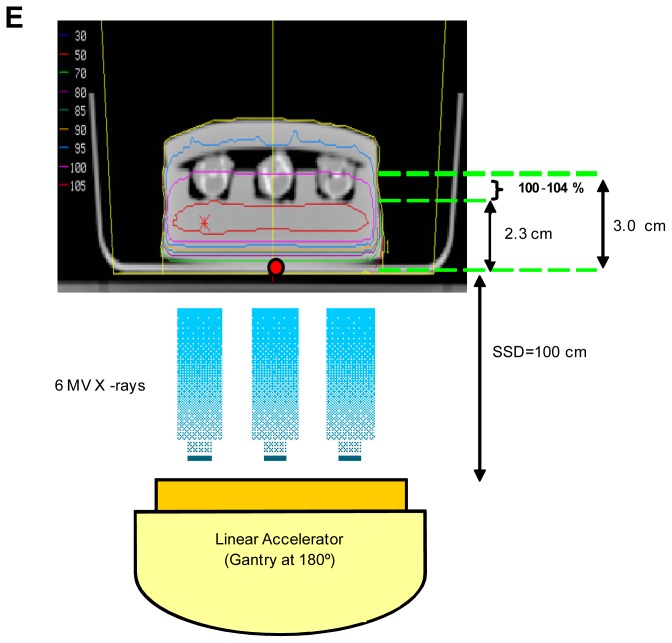
Standardization of the experimental radiation model box. (**A**) The molded bolus was placed in the plastic box. Black lines are drawn on the four sides of the box indicating the position of the bolus. In the treatment room, laser lines used for alignment (red brackets) pass through the black lines to ensure good and reproducible alignment throughout the treatment; (**B**) Anesthetized mice laid on the bolus surface in supine position, with their heads fitted in the molded beds and their bodies kept in position by means of sticking-plaster; (**C**) Placement of bolus with fixed animals according to the position of the black marks. Yellow rectangle indicates the projection of the treatment field size and the red crossline the direction of the alignment laser lights on the treatment table; (**D**) Other molded bolus covering the heads of the mice for CT scanning and treatment. (**E**) The depths at which brains are placed with regard to the box surface (between 2.3 and 3.0 cm depth) and the isodose curve distribution at these depths. SSD: Source Surface Distance = 100 cm. The figure also shows how the brains are irradiated.

Radiation therapy is the most commonly used clinical procedure for the treatment of brain metastases. We aimed to standardize an experimental radiation model for application in preclinical radiation therapies. We used a scalpel to mold several beds on the bolus (one for each mouse’s head) in order to keep all the heads in the supine position during CT scanning and treatment. When mice were anesthetized in the flux chamber, they were laid on the bolus surface in the supine position, with their heads fitted in the molded beds after securing their bodies in position with sticking-plaster (Figure 3A–E). A piece of molded bolus covered their heads; care was taken not to disturb the breathing.

After the head CT scans were performed, they were processed by the planning software by calculating the dose distribution (isodose curves) of ionizing radiation in brain tissue from the CT slices. This procedure provided precise information about the relevant physical parameters, the dose distribution, the energy value of the chosen X-rays and prescription data (Figure 3E). The radiation treatment schedule was then planned to ensure that the dose delivered to the brain was always the same throughout all the treatment sessions.

For radiobiological assessments in radiotherapy, the linear–quadratic model (L–Q model) is widely used [30]. This mathematical model provided a way to calculate the change of fractionation between two treatment schedules needed to maintain the same isoeffect in both the tumor and healthy tissue, taking into account the number of fractions and the dose per fraction. The balance between antitumoral efficacy and tolerance in healthy tissues permitted the assessment of the isoeffect in different treatment schedules. We selected the schedule based on 5.5 Gy/fx × 1 fx/day × 3 days (overall dose: 16.5 Gy) firstly because the dose delivered to the healthy brain was smaller than those delivered with the 3 Gy/fx × 1 fx/day × 10 days schedule (overall dose: 30 Gy), reducing the risk of early death by brain toxicity, and secondly because the treatment period was shorter and animals received intraperitoneal anaesthesia for only three consecutive days.

The experimental protocol was accordance with the role of whole brain radiotherapy (WBRT) in preventing brain metastasis. In humans, the treatment of choice for brain metastasis in most cases is WBRT, regardless of the histological type. One of the standard schedules is 3 Gy/fraction, delivering one fraction/day for ten days, with an overall delivered dose (OD) of 30 Gy (3 Gy/fx × 1 fx/day × 10 day). We tested this treatment schedule in several preliminary studies by delivering it to the whole brain of mice with variable degrees of immunodeficiency. First, we checked the survival of CD1 and Nude Balb/c female mice exposed to this schedule, and found that CD1 mice survived more than five months without clinical signs of brain damage or loss of weight; only corneal opacity was observed. The Nude Balb/c female mice treated with the same protocol died two months after the radiation therapy (data not shown).

The treatment schedule was performed in brains of female Athymic Nude-Foxn1nu mice at a depth of between 2.3 and 3.0 cm from the bolus surface. Taking into account the balance between efficacy/tolerance, one fraction (5.5 Gy) was delivered for three days (overall dose: 16.5 Gy); the dose rate was 240 Monitor Units (MU/min). The results showed improved survival of mice treated with radiotherapy with regard to controls (60.29 ± 8.65 *vs.* 47.20 ± 11.14, Kaplan-Meier, Log Rank: Chi-Square 2.456; df = 1; *p =* 0.117).

The apparent differences between BRV5 and BRV5CA1 controls were not significant (Kaplan-Meier. Log-Rank test; *p* = 0.228) and may have been due to the biological variability through *in vivo* experiments and the more aggressive phenotype obtained with BRV5CA1 cells.

These schedule based on a 5.5 Gy/fraction delivered in one fraction/day for three days, with an overall dose of 16.5 Gy was tested in several kinds of immunodeficient mice. Female athymic mice survived at least six months after radiotherapy without loss of weight, while NOD/SCID female mice died within eight days of radiotherapy which induced extreme weight loss (data not shown). This evidence suggests that radiosensitivity is dependent on the mouse model used, particularly on the immunological status of mice.

### 2.3. Combined Irradiation and Chemotherapy to Treat Experimental Brain Metastasis

**Figure 4:**
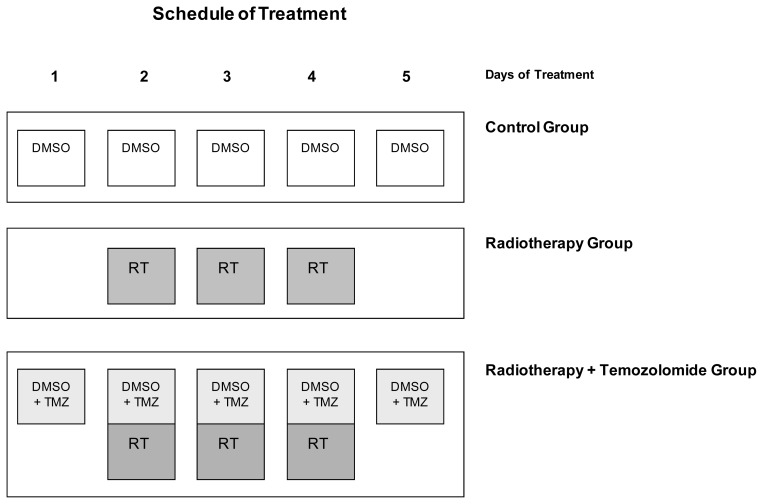
Schedule of treatment for each group of study: dimethyl sulfoxide (DMSO) vehicle alone for control group (*n* = 5); radiotherapy alone for radiotherapy group (*n* = 7); and concomitant chemoradiotherapy for radiotherapy plus temozolomide (TMZ) group (*n* = 9). The first day of treatment was 26 days after *IC* injection of cells when bioluminescence analysis showed brain metastasis growth. The treatment was applied on five consecutive days.

Most clinical trials for brain metastasis enroll patients who have progressed after WBRT treatment, or test the therapy in combination with WBRT. In order to optimize the therapeutic model, we performed experiments combining radiation with radiosensitization by the DNA methylation agent temozolomide, which is currently undergoing clinical evaluation for cancer therapy [6]. As temozolomide has been shown to increase survival rates of patients with malignant gliomas when combined with radiation [31], we investigated its possible enhancing effect on radiosensitivity (Figure 4).

The TMZ dose was selected on the basis of previous studies on glioma models using TMZ at a dose of 100 mg/Kg/day delivered intraperitoneally [32]. Since other tumour xenograft model studies evaluated TMZ at 200 mg/Kg/week (100 mg/Kg/day for two days a week) intragastrically for several weeks, the maximum tolerated dose was not studied. Moreover, in humans a reduction in the daily dose of TMZ is needed when the drug is delivered concurrently with radiotherapy due to TMZ’s concomitant toxicity [33]. Thus, in our model, in which TMZ was administered concomitantly with radiotherapy, we decided to use a reduced dose of TMZ (60 mg/Kg/day) to decrease the risk of death due to toxicity.

**Figure 5:**
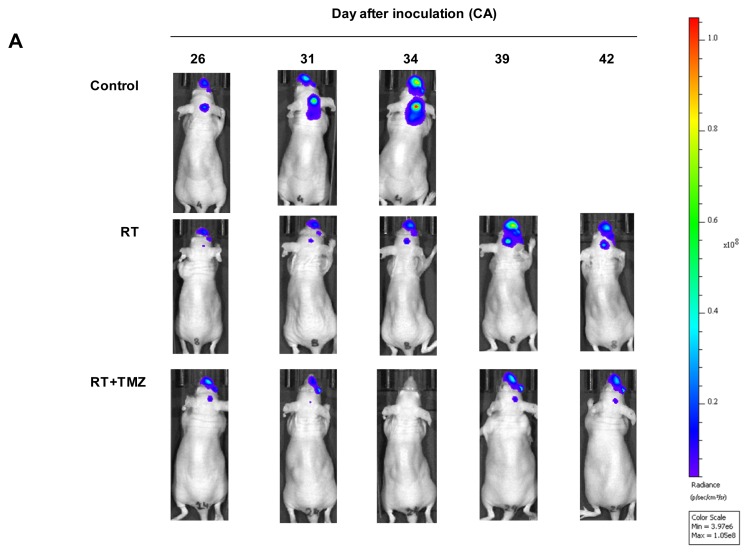

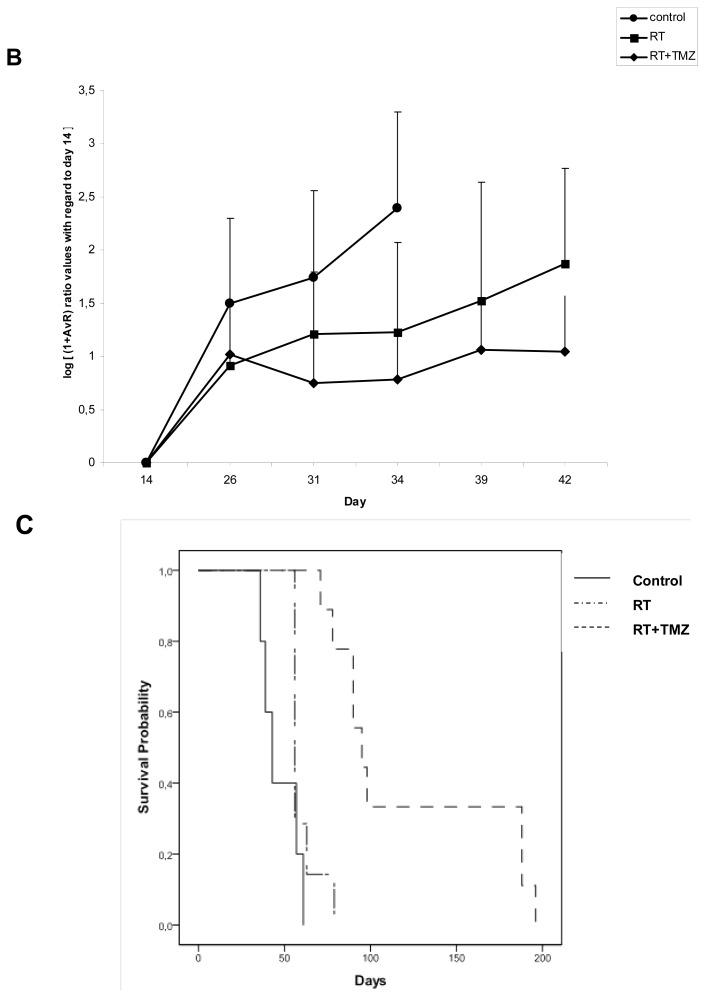
Combined irradiation and chemotherapy to treat brain metastasis. (**A**) Control mice, mice treated with radiotherapy and mice treated with chemoradiotherapy were compared from day 26 to 42 after injecting cells in *CA*. The evolution of metastasis growth is shown according the signal of luminescent cells; (**B**) The median of metastatic burden of mice treated with radiotherapy and treated with chemoradiotherapy was compared from the starting point of the induction of metastasis; (**C**) The survival evolution is indicated in the Kaplan-Meier Curve (Log Rank -Mantel-Cox-: Chi-Square 22.022; df = 2; *p <* 0.001). Control *vs.* RT: Log Rank (Mantel-Cox): Chi-Square 2.456; df = 1; *p =* 0.117. RT *vs.* RT + TMZ: Log Rank (Mantel-Cox): Chi-Square 13.669; df = 1; *p <* 0.001. Control *vs.* RT + TMZ: Log Rank (Mantel-Cox): Chi-Square 16.649; df = 1; *p <* 0.001.

Mice began the treatment on day 26 after brain metastases were induced, when the follow-up bioluminescence indicated metastasis development. The control group received DMSO solution orally on five consecutive days. Radiotherapy (cranial irradiation 5.5 Gy) was administered on three consecutive days starting on the 27th day after the *IC* injection of cells. In addition, DMSO + TMZ solution was delivered orally from day 26 post-*IC* inoculation of cells on five consecutive days. On days 2–4 TMZ was administered one hour before cranial irradiation (cranial irradiation 5.5 Gy), as described in the protocol (Figure 5).

Brain metastasis evolution in mice treated with 5.5 Gy/fraction, delivered one fraction/day for three days (days 2–4, overall dose: 16.5 Gy), were compared with mice treated with radiotherapy plus chemotherapy (RT + TMZ), which received 20 μL DMSO + TMZ and 180 μL saline orally (final volume delivered: 200 μL), every day for five days (days 1–5) and 5.5 Gy/fraction, delivered one fraction/day for three days (days 2–4, overall dose: 16.5 Gy). Brain metastases were followed by luminescence and the evolution of the treated groups was compared with that of a control group which received the vehicle orally (Figure 5A, Table 2). Indeed, 42 days after starting the treatment differences were recorded in brain metastasis burden between RT alone and RT + TMZ (Figure 5B). By the end of the experiment survival was significantly higher in the RT + TMZ group (121.56 ± 52.53 days) than in the RT alone group (60.28 ± 8.65 days), RT *vs.* RT + TMZ, Log Rank: Chi-Square 13.669; df = 1; *p <* 0.001. These results demonstrate the benefit of chemoradiotherapy for treating breast cancer brain metastasis (Figure 5C).

## 3. Discussion

Metastasis research is highly dependent on reliable animal models that allow different aspects of metastasis initiation and progression to be studied *in vivo*. As these pathologies cannot be studied longitudinally in humans for evident ethical reasons, *in vivo* follow-up studies require animal models that can accurately reflect the process observed in humans [16,17]. To develop a more efficient model of brain metastasis from advanced breast cancer we used 435-Br1 brain metastatic cells, established from brain metastasis in nude mice, which has been functionally characterized elsewhere [34]. Thus, by injecting cells into the *LV* and recover them from the brain metastasis we increased specificity due to the selection of cells that are able to cross the blood brain barrier and to adapt to the brain microenvironment.

The BRV5CA1 cells injected via the IC induced histologically confirmed metastases in 100% of animals, but no large masses developed either in the jaw or the neck. Moreover, in our study, bioluminescence images showed cranial signals in 19/23 animals (83%) on day 26 post-injection (just before the start of the treatment protocol). These results significantly improve the brain metastasis evolution of 435Br1 cells, since in our previous magnetic resonance imaging (MRI)/spectroscopy (MRS) characterization [21] brain lesions were localized in different brain regions in 69% of animals, which were further histologically confirmed in 61% of cases. Since other metastases appeared in the neck and the right jaw, progressing rapidly and causing feeding problems in mice, the global procedure was inefficient. The selection procedure through five *in vivo/in vitro* passages of 435Br1 cells induced BRV5CA1 cells with a better efficiency and specificity for developing clinical brain metastasis.

It is known that arrest of individual tumor cells in brain capillaries induces diverse astrocytic and microglial responses, resulting in heterogeneous local changes of the initial tumor microenvironment, which in turn could restrict the progression of metastatic cells into macrometastases [29]. The survival of arrested cancer cells within brain capillaries may be a rate-limiting step in metastatic progression, since cancer cell penetration of the vessel wall in the brain is much slower than in progression and impacts the success of tumor cells to survive and grow within the brain [35–37].

The current incidence of brain metastasis seems to be the paradoxical result of the effectiveness of drugs that do not cross the blood brain barrier (BBB). In this study of the effectiveness of an experimental approach combining radiation and chemotherapy we selected temozolomide, an alkylating agent which to our knowledge has not been fully introduced in treatment protocols for breast cancer brain metastasis although in patients with brain metastatic breast cancer no responses have been reported when was used as a single agent [38]. Different response degrees have been reported in patients with melanoma or non-small cell lung cancer brain metastasis treated with TMZ concomintant with whole brain radiation [39–41]. Further studies are needed to demonstrate the benefits of TMZ concomitant with radiation therapy on breast cancer brain metastasis.

Trials with TMZ as a radiosensitizer to treat breast cancer brain metastasis are underway [42]. The treatment schedule is based on whole brain radiotherapy at 3 Gy/day administered over a two-week period and an induction with TMZ 75 mg/m^2^/day during this period, following TMZ 750 mg/m^2^ (fractionated). Moreover, in a phase II study, Siena, *et al.*[43] reported a dose-dense temozolomide regimen for treatment of brain metastasis in which patients had median progression-free survivals of 56, 58, and 66 days for melanoma, breast cancer, and non-small cell lung. New clinical trials are underway to supplement the action of temozolamide with pegylated liposomal doxorubicin, showing an overall response rate of 36.8% and a significant improvement in quality of life [44]. Furthermore, drug pharmacokinetics and biodistribution may be increased using nanocarriers to enhanced drug delivery into the brain improving drug accumulation [45,46].

The role of systemic chemotherapy other organs [47]. Moreover, it is well established that the host microenvironment affects metastasis in patients with brain metastases remains unclear [48]. Treatment of brain metastasis in breast cancers includes surgery and radiation therapy, since the efficacy of chemotherapy for brain metastases remains disappointing [49]. In clinical studies, particularly in non-small-cell lung cancer radiotherapy reduced the incidence of brain metastasis, but did not achieve any substantial survival benefit [16,50]. Although reported response rates range from 56% to 82% in patients with primary cancer of the lung and breast the associated adverse events are severe [50,51]. Clearly, a chemotherapeutic agent that is both efficacious and well tolerated would hold great potential for the treatment of patients with brain metastases from solid tumors [52].

Most clinical trials for brain metastases enroll patients with diagnosed brain metastasis and test an experimental therapy in combination with WBRT. Little effect on patient survival has been achieved. This experimental chemoradiotherapy model of brain metastasis provides a reproducible method for checking new drugs and can help to establish new therapeutic protocols.

## 4. Experimental Section

### 4.1. Cells and Primary Cultures

435-Br1 human mammary carcinoma cells (originally established from a brain metastasis in a *nude* mouse orthotopically inoculated with the triple negative parental cell line, MDA-MB-435, kindly supplied by Dr. Fabra, IDIBELL, in 1992) were maintained under standard conditions in 1:1 (*v*/*v*) mixture of DMEM and Ham F12 medium, DMEM/F12 (Life Technologies, Inc. Gibco BRL, Gaithersburg, MD, USA). This mixture was supplemented with 10% fetal bovine serum (FBS), 1 mM pyruvate, and 2 mM l-glutamine in a 5% CO_2_ environment at 37 °C in a humidified incubator [9]. Although a controversial point, it has recently been demonstrated that MDA-MB-435 cells represent a useful breast cancer model and that they express both epithelial and melanocytic markers [53].

435-Br1 cells in exponential growth phase were first treated with Trypsine-EDTA (Life Technologies, Gaithersburg, MD, USA) for 1 min at room temperature for inoculation. They were then washed twice in HBSS and counted using a Neubäuer chamber. Viability (measured by Trypan-Blue exclusion) was always between 90% and 97%. 435-Br1 cells were then resuspended in HBSS (Hanks’ Buffer Saline Solution) to obtain a final concentration of 1 × 10^6^ cells/100 μL for inoculation either into the left ventricle (*LV*) or into the internal carotid artery (*CA*) of mice, using a slight modification of a previously described method [21].

Primary cultures were performed in DMEM F-12 with 50% FBS (Fetal Bovine Serum) (*v*/*v*), 2% l-glutamine plus pyruvate (*v*/*v*), Penicillin 1 mg/mL, Streptomycin 1 mg/mL, Neomycin 2 mg/mL (from antibiotic PSN 100× solution, Gibco, Gaithersburg, MD, USA), Gentamicin 0.2 mg/mL and Amphotericin-B (Fungizone) 2.5 μg/mL. After culture, if cells showed optimum growth, we reduced FBS to 20% and gradually removed the antibiotic solution. Finally, we worked with the standard medium DMEM F-12 10% FBS with l-glutamine and pyruvate without bacterial or fungal contamination. Mycoplasma-free status was checked before preparing the cells for the next inoculation, in order to obtain metastatic cells with a greater affinity to seed in the mouse brain after five cycles of inoculation in *LV*.

### 4.2. Retroviral Transduction

To label 435-Br1 brain metastatic cells we used the retroviral vector preGFP-CMV-PLuc, which contained the enhanced green fluorescent protein (*eGFP*) gene, under control of the 5′ LTR, and the photinus luciferase (*PLuc*) gene, under control of the cytomegalovirus (CMV) promoter. Retroviral transduction was used to label 435-Br1 brain metastatic cells. Vector preparation and packaging of viral particles was performed as described previously [54]. A cell population that uniformly expressed the highest levels of eGFP (BR-eGFP-CMV/Luc) was selected by FACS (MoFlo, Cytomation, Dako, Denmark).

### 4.3. Brain Metastasis Model and *in Vivo* Experiments

Athymic Nude-Foxn1nu female mice 22–28 g weight were purchased from Charles-River Laboratories (Wilmington, MA, USA) and were housed at the IDIBELL facility in SFP conditions, with 20–24 °C cage temperature, 60% relative humidity, and 12–12 h light-dark periods. Animals were allowed free access to UV irradiated water and an adequate sterile diet. All animal-related procedures were performed in accordance with the National Institute of Health Guidelines for the Care and Use of Laboratory Animals, with the approval of the animal care committee.

Briefly, mice were anesthetized with ketamine and xilacine solution (ketamine: 100 mg/Kg; xilacine: 10 mg/Kg) injected intraperitoneally before to inoculate cells into the *LV*. The procedure was performed under sterile conditions in a flux chamber and after cleaning the skin with antiseptic iodine solution. The ribs were visualized by opening the skin lengthwise at the left parasternal line, and 1 × 10^6^ BR-eGFP-CMV/Luc cells/100 μL HBSS were injected between the third and fourth ribs perpendicularly to the chest surface. Finally, we closed the skin with staples. Animals were sacrificed by means of intraperitoneal injection of sodium pentobarbital (Dolethal^®^), according to their weight, when clinical signs of illness or weight loss were detected. The brain and other organs such as lungs, liver, suprarenal glands, ovaries, mediastinal and abdominal lymph nodes were then removed and examined under a fluorescent microscope for the presence of BR-eGFP-CMV/Luc cells. Cells isolated from brain tissue underwent primary culture processes. *LV* injection of cells and their further isolation from mouse brain was repeated five times, obtaining BR-eGFP-CMV/Luc-V1 to V5 cells through these cycles.

To induce brain metastases by *CA* inoculation, supine mice were anesthetized with inhalatory anesthesia under sterile conditions in a flux chamber using a mixture of O_2_ and isofluorane at 5% (with a flux of 4 L/min) for induction, and isofluorane at 1.5%–2.0% (with a flux of 0.3 L/min) for maintenance. The whole neck was then disinfected with antiseptic iodine solution and glucosaline *s.c.* (20 mL/Kg per day for two days) was injected to maintain good hydration and meloxicam 5% *s.c.* (100 μL/20 gr per day for two days), to achieve an optimum analgesic/anti-inflammatory effect. The neck skin was then opened with an incision like an “inverted seven” (first lengthwise and then sideways to the right), thus obtaining a wide surgical field. All soft tissues were dissected, releasing the structures carefully to identify clearly the right carotid artery with its main branches. Then, we clamped (Vascular Clamps F.S.T. ref.00396-01–S&T, Switzerland) the root of the external carotid artery, under the origin of its occipital branch, and then the stapedial branch, located cranially and deep in the neck. In this way we ensured that most of the inoculated cells (1 × 10^6^ of BR-eGFP-CMV/Luc (V5) cells/100 μL HBSS) with a Hamilton^®^ syringe (100 μL Bonaduz, Switzerland) and a Hamilton^®^ needle (33 Gauge, PK6) would slowly access the brain through the internal carotid artery. Using a pair of tweezers, we grasped the common carotid artery caudally near the base of the neck and maintained it immobile during the injection. The injection point of the cellular suspension was chosen just above this site. Once the needle was inside the common carotid artery lumen, we inserted it in the internal carotid branch lumen and then delivered the cellular suspension, pushing slowly at the point of injection until the bleeding stopped. Finally, we finished applying the iodine solution in the surgical bed and clamped the skin by means of staples. We then stopped the isofluorane mixture and delivered a high concentration of O_2_ for a few seconds before placing the mice under a dry hot source.

Animals were sacrificed by means of intraperitoneal injection of sodium pentobarbital (Dolethal^®^) according to their weight when clinical signs of illness or loss of weight were detected.

BR-eGFP-CMV/Luc-V5CA cells were inoculated into *CA* of mice for the chemoradiotherapy experiment.

### 4.4. *In Vivo* Set-up

Athimic mice inoculated with BR-eGFP-CMV/Luc (V5CA) cells in right carotid artery were explored with magnetic resonance imaging sequences in a high-field (7T) horizontal spectrometer at 29 days post-injection. These studies were carried out at the joint NMR facility of the Universitat Autònoma de Barcelona and CIBER-BBN (Cerdanyola del Vallès, Spain), using a 7 T horizontal magnet (BioSpec 70/30; Bruker BioSpin, Ettlingen, Germany) equipped with actively shielded gradients (B-GA12 gradient coil inserted into a B-GA20S gradient system) and a quadrature receive surface coil, actively decoupled from a volume resonator with 72 mm inner diameter.

Anesthesia was performed using isoflurane and O_2_ mixture (2.5%–3.0% for induction, and 0.7%–2.5% for maintenance). Respiratory frequency was maintained between 40 and 60 breaths/min. Body temperature was maintained between 36.5 and 37.5 °C with a recirculating water system incorporated in the animal bed, and measured with a rectal probe. Breathing rate and temperature were constantly monitored (SA Instruments, Inc., New York, NY, USA). All animals were explored in coronal and axial planes with T2-weighted high resolution MR images. For this, a RARE sequence was chosen; field of view (FOV), 19.2 × 19.2 mm; matrix (MTX), 256 × 256 (0.075 × 0.075 mm/pixel); number of slices (NS), 10; slice thickness (ST), 0.50 mm; echo time (TE), 12 ms (effective TE, 36 ms); recycling time (TR) 4.2 s; number of averages (NA), 4; total acquisition time (TAT), 6 min 43 s.

### 4.5. Bioluminiscence Analysis

Inhalatory anesthesia with O_2_ and isofluorane mixture was delivered to athymic mice before image acquisition: induction was performed outside the bioluminiscence chamber (isofluorane 4% at 2 L/min) and maintenance inside the chamber (isofluorane 2% at 2 L/min) during acquisition. Animals were placed in prone position. The photons recorded in the images were quantified and analyzed using Living Image 4.1 image analysis software (Caliper, LifeSciences Hopkinton, MA, USA). The number of photons was expressed as photon counts per second (p/s). The parameter chosen for treatment evaluation was the Average Radiance (p/s/cm^2^/sr). Luciferin solution (d-Luciferin Firefly potassium salt, l-8220–Biosynth AG) was prepared according to the data sheet. Intraperitoneal injection of luciferin solution (200 μL/20 g) was applied 10 min before imaging. The planned period of time for image acquisition was the same for all animal groups (control, radiotherapy and radiotherapy plus temozolomide) before and after treatment.

Background signals were subtracted from all the bioluminescence cranial measurements as part of image analysis. To this end, a healthy mouse (not inoculated with tumoral cells) received luciferin solution (200 μL/20 g) intraperitoneally 10 min before imaging.

### 4.6. Histological Analysis

During the organ-specific selection procedure, after the mice had been sacrificed we examined the presence of green fluorescent cells in lungs, liver, suprarenal glands, ovaries, lymph nodes and brain under fluorescent microscopy. Finally, soft tissues were fixed with formalin and embedded in paraffin. Brains were optionally fixed in 4% paraformaldehyde 24 h, followed by 30% sucrose 24 h and OCT-embedded (tissue freezing medium, Sakura Tissue-Tek^®^) before being frozen in dry ice. Samples, which were kept at −80 °C, were cut into 5 μm coronal sections for evaluation. The cryostat was at −27 °C.

Metastatic involvement was explored in each section by classic hematoxylin-eosin (H&E).

### 4.7. Therapeutic Protocols

We used a combination of radiotherapy and chemotherapy to standardize a preclinical model which mimicked the clinical situation of brain metastasis development, in order to assess the efficacy of both treatments.

### 4.8. Radiotherapy

Under sterile conditions in a flux chamber, we anesthetized mice by intraperitoneal injection of ketamine (100 mg/Kg) and xylazine (10 mg/Kg). For irradiation mice were transported in a hermetically closed plastic box from the flux chamber to the scanner, where CT slices were performed in the head placed in a helical CT scanner device (Oncology Radiotherapy Service of the Catalan Oncology Institute, ICO, Hospital Duran i Reynals, L’Hospitalet de Llobregat, Spain), and then to the radiation treatment unit. A flexible and solid silicone gel, Bolus (Lorca Marín, S.A., 30007-Murcia. Spain), which has the same density as the body tissues, was used to obtain a reliable dose distribution of ionizing radiation at the prescribed depth.

We used the Cadplan Treatment Planning Software (Varian dosimetry software, Darmstadt, Germany), to calculate the dose distribution (isodose curves) of ionizing radiation from the CT slices (Servei de Física Mèdica, ICO, L’Hospitalet de Llobregat, Spain). All treatment sessions were performed with a Linear Accelerator CLINAC-2100 device (Varian Oncology Systems, Darmstadt, Germany) for radiotherapy at the Oncology Radiotherapy Service, which allowed us to deliver 6 MV X-ray energy (SOR, ICO, L’Hospitalet de Llobregat, Spain).

### 4.9. Chemotherapy

Mice were treated with temozolomide (TMZ), a novel oral alkylating agent with proven clinical activity in primary and recurrent gliomas and metastatic melanoma [32,55,56]. 100 mg TMZ (≥98% HPLC, solid T2577, Sigma-Aldrich Química, St. Louis, MO, USA) were resuspended in 0.5 mL of dimethyl sulfoxide (DMSO, D5879 Sigma-Aldrich, St. Louis, MO, USA) as vehicle with a final concentration of 200 mg/mL. To achieve homogeneity the solution was sonicated (UP50H Ultrasonic Processor, Hielscher, Ultrasound Technology, Teltow, Germany), delivering four 5 s pulses (each one with a 30% amplitude) every five seconds. Aliquots were frozen at −80 °C.

Mice were orally administered 60 mg/Kg/day in a 200 μL final volume (20 μL of TMZ + DMSO solution and 180 μL of saline) using a flexible and sterile intragastric catheter (Instech Solomon, Plymouth, PA, USA). The control group comprised mice orally administered 20 μL DMSO and 180 μL saline solution every day for five days (days 1–5).

### 4.10. Statistics

For survival times for V3 (LV) and V5 (CA) groups, we used the non-parametric Mann-Whitney test.

The bioluminescence data were transformed using the log(1 + *x*) function (where *x* = AvR), in order to obtain a more regular and positive distribution. Subsequently, these data were normalized by subtracting the first observation (day 14) from each of the following ones. The Student *t* test was used to compare the treatment groups. Survival curves for each treatment were estimated via the Kaplan-Meier method, and the Log-Rank test was used to assess if they were significantly different.

*p*-values lower than 0.05 were considered significant.

## 5. Conclusions

In conclusion we have generated a new breast cancer brain metastatic cellular model, which induced parenchymal brain metastasis within a 60-day time window post-injection in carotide in all cases. This versatile model mimics clinical brain metastasis growth and can be analyzed *in vivo* when mice are submitted to therapeutic protocols. Moreover, we have optimized a new preclinical chemoradiotherapy protocol proved useful for the study of radiation response/resistance in brain metastasis, either alone or in combination with new sensitizing agents.

## Figures and Tables

**Table 1:** Distribution of organs with progressive metastatic colonization in BRV3 *vs.* BRV5 cells inoculated mice.

	Via left ventricle	Via internal carotid artery
		
	V3 (*n* = 5)	V5 (*n* = 5 )
		
	positive GFP-Luc cells	positive GFP-Luc cells
Brain	2/5 (40%)	5/5 (100%)
Lungs	5/5 (100%)	5/5 (100%)
Liver	4/5 (80%)	1/5 (20%)
Suprarenal glands	1/5 (20%)	3/5 (60%)
Ovaries	2/5 (40%)	2/5 (40%)
Mediastinic Lymph Nodes	5/5 (100%)	3/5 (60%)
Abdominal Lymph Nodes	1/5 (20%)	1/5 (20%)

**Table 2:** Comparison of controls, treated with radiotherapy alone or in combination with chemotherapy.

Day	26	31	34	39	42
control *vs.* RT	0.2039	0.2348	**0.0526**	-	-
control *vs.* RT+TMZ	0.2639	**0.0552**	*0.0115*(*)	-	-
RT *vs.* RT+TMZ	0.7024	0.1813	0.3024	0.3337	**0.0589**

* *p*-value is obtained according the median metastatic burden in the brain in each group: controls *N* = 5, RT *N* = 7 and RT + TMZ *N* = 9 (Student “*t*” test; 2-tailed).
